# Quality of life in childhood epilepsy with lateralized epileptogenic foci

**DOI:** 10.1186/1471-2377-10-69

**Published:** 2010-08-17

**Authors:** Krystyna A Mathiak, Małgorzata Łuba, Klaus Mathiak, Katarzyna Karzel, Tomasz Wolańczyk, Elżbieta Szczepanik, Paweł Ostaszewski

**Affiliations:** 1 Department of Child and Adolescent Psychiatry and Psychotherapy, RWTH Aachen University, Germany; 2 Faculty of Psychology, University of Warsaw, Poland; 3 Department of Psychiatry and Psychotherapy, RWTH Aachen University, Germany; 4 INM-1, Forschungszentrum Jülich GmbH, Germany; 5 Department of Child Psychiatry, Medical University of Warsaw, Poland; 6 Clinic of Neurology of Children and Adolescents, Institute of Mother and Child, Warsaw, Poland

## Abstract

**Background:**

Measuring quality of life (QOL) helps to delineate mechanisms underlying the interaction of disease and psychosocial factors. In adults, epileptic foci in the left temporal lobe led to lower QOL and higher depression and anxiety as compared to the right-sided foci. No study addressed the development of QOL disturbances depending on the lateralization of epileptogenic focus. The objective of our study was to examine QOL in children with lateralized epileptiform discharges.

**Methods:**

Thirty-one parents of children with epilepsy filled the Health-Related Quality of Life in Childhood Epilepsy Questionnaire (QOLCE). Fifteen children had foci in the left hemisphere and sixteen in the right, as verified with Electroencephalography (EEG) examinations.

**Results:**

We found a significant correlation between foci lateralization and reduced QOL (Spearman's rho = 0.361, p < 0.046). Children with right hemispheric foci exhibited lower overall QOL, particularly in five areas: anxiety, social-activities, stigma, general-health, and quality-of-life.

**Conclusions:**

We demonstrated for the first time that in children left- and right-hemispheric foci were associated with discordant QOL scores. Unlike in adults, foci in the right hemisphere led to worse emotional and social functioning demonstrating that seizures impact the brain differentially during development.

## Background

Quality of life (QOL) is an important outcome measure in clinical research, taking into account patient's subjective evaluation of his own being and supplementing the traditional medical approach, based on the examination of symptoms, signs, and laboratory findings as defined from the doctor's perspective [1]. World Health Organization (WHO) defined QOL as 'individuals' perception of their position in life in the context of the culture and value systems in which they live and in relation to their goals, expectations, standards and concerns' [2]. The term incorporates multidimensional aspects of individuals' physical health, psychological state and social relationships. Quality of life is an important measure in epilepsy, which is often a chronic and debilitating condition and unique among the chronic illnesses due to the multidimensional impact on psychosocial functioning [3].

Childhood epilepsy is among the most prevalent and important neurological conditions in the developing years, affecting 3.6 to 4.2 per 1000 children in developed countries, and approximately double these rates in developing countries (for a review, see [4]). The consequences of epilepsy often evolve in a complex fashion and can be accounted for by a number of factors including the effects of brief but chronic dysfunctional episodes on the developing brain, the presence of comorbid disabilities, the subsequent cognitive problems, and the reaction of the individuals and their families to the diagnosis and treatment [5]. The improvement of clinical parameters only, even including remission, does not need to lead to the improvement of social functioning and subjective QOL [6,7].

The unique feature of epilepsy, among other chronic conditions, is its direct effect on the brain. This might contribute to more disturbed psychosocial functioning in patients diagnosed with epilepsy as compared to other chronic, stigmatizing disorders. Therefore our understanding of psychological and social effects of epilepsy can be enhanced by neuropsychological insights into the brain-behavior relationship [8]. Studying behavioral consequences of lateralized potentially epileptogenic foci may offer an insight into understanding these links.

Lateralized seizures have differential impact on behavior. It was observed that right temporal lobe seizures evoke experiential phenomena more often, including an intense sense of the familiar and unfamiliar, fear, and complex multisensory hallucinations [9]. Left-sided foci may lead to ictal outbursts of laughing (gelastic epilepsy) [10]. Depression and anxiety were typically observed in patients with left- versus right-sided temporal lobe epilepsy [11,12]. Andelman et al. [13] demonstrated that left temporal epilepsy is associated with higher level of anxious personality trait and a decreased self-assessment of QOL as compared to right-sided focus. A number of studies have demonstrated that lateralization patterns may change over the developmental period (for a review, see [14]). Upton and Thompson [15] showed that frontal epileptic focus has differential effect on cognitive functions in children, depending on age when the disease started. Their finding demonstrates that lateralized seizures impact behavior differently depending on age.

Although early epidemiological studies suggest that left temporal lobe epilepsy in children is related to increased behavioral problems, in particular hyperactivity, antisocial behavior and aggression, this finding has not been confirmed so far (for a review, see [16]). Cohen et al. [17] found disturbed prosody recognition in children with right-sided focus only, therefore demonstrating that in the developmental age lateralized seizures can influence neuropsychology differentially. Mathiak et al. [18], using generic scales, demonstrated higher level of disruptive behavior in children with right sided foci and more internal locus of control in children with left-hemispheric seizures. To assess complex multidimensional consequences of epilepsy, a broad assessment of QOL was recommended [19]. Studies of QOL in unilateral epileptic seizures have concentrated exclusively on adults.

The Health-Related Quality of Life in Childhood Epilepsy Questionnaire (QOLCE; [20]) examines QOL in a wide range of life domains including physical, cognitive, emotional, and social functioning and was developed and validated specifically for children with epilepsy. Such scales were shown to have higher sensitivity to disturbances in epilepsy than generic instruments [21]. Our objective was to assess whether there was an association between lateralized epileptiform discharges in childhood epilepsy and QOL. Children with right and left epileptiform discharges completed QOLCE scale. We hypothesized that foci lateralization correlates with a reduced QOL in children with unilateral epilepsy thus confirming a direct brain-behavior interaction influencing QOL during development.

## Methods

### Subjects

A group of 51 children from the Institute of Mother and Child in Warsaw and the Warsaw Hospital for Children was recruited based on hospital discharge cards. Patients were included in the presence of (1) unilateral epileptic focus as determined by presence of epileptiform activity in EEG examinations, (2) age in the range of 6-15 years, (3) normal intelligence (IQ above 85 in Wechsler's scale), and (4) no accompanying chronic diseases that was not a direct outcome of epilepsy, such as asthma or diabetes, or any disease that started before the onset of epileptic seizures. We included all the children meeting the inclusion criteria who visited the outpatient clinic during the time of the study and over 90% of them agreed to participate. The exclusion criteria were: (1) generalized discharges in an EEG or unclear lateralization; changing lateralization patterns in consecutive EEG examinations, (2) first epileptiform discharges which had occurred less than one year prior to the examination, or (3) anatomical malformations detected by cranial computed tomography or magnetic resonance. Twenty patients were excluded: 11 subjects had bilateral or generalized discharges; in four the disease was diagnosed less then one year earlier, and five had anatomical findings. Finally a group of 31 children (12 girls and 19 boys, mean age11.2 ± 2.7, 16 with right and 15 with the left hemispheric focus) took part in the study. Patient characteristics are summarized in Table 1. Prior to the study, a written informed consent was obtained from a caregiver as well as an oral consent from the child. The Ethics Committee of the Institute for Mother and Child in Warsaw, Poland approved the study protocol.

**Table 1:** Individual clinical characteristics.

Age (years)	Sex	Disease (years)	Medication (years)	Antiepileptic drugs at the time of examination*		Hospitalizations per year	Number of seizures per month
Left-hemispheric seizure focus							

7	Boy	1	1	1	C	1.00	< 1

8	Boy	1	1	3	C V L	2.00	1 - 9

8	Boy	5	5	2	C L	0.50	1 - 9

9.5	Girl	5	3	1	C	0.20	1 - 9

10.5	Boy	2	1	1	O	1.00	1 - 9

11	Boy	4	4	3	V T Cl	1.00	1 - 9

11	Boy	6	5	2	C L	0.50	1 - 9

11.5	Boy	3	3	1	L	0.50	> 30

12	Girl	10	8	1	O	0.25	> 30

12	Girl	8	2	1	O	0.18	> 30

13	Girl	3	3	1	O	0.50	1 - 9

13	Girl	10	9	3	V L T	0.67	< 1

13	Boy	3	2	1	O	0.20	> 30

13.5	Girl	5	5	1	V	0.80	< 1

14.5	Boy	3	0	1	V	0.00	1 - 9

15	Boy	4	4	1	O	0.33	1 - 9

Right-hemispheric seizure focus							

6	Girl	3	2	2	C V	0.33	< 1

6	Boy	3	1	2	O V	0.33	1 - 9

7	Girl	1	1	3	O V T	1.00	> 30

8	Boy	1	1	3	V T Et	3.00	> 30

9.5	Girl	6	2	1	V	0.17	10 - 30

10.5	Boy	8	2	1	C	0.33	< 1

10.5	Girl	3	2	1	O	0.25	< 1

11.5	Boy	4	4	1	C	0.13	1 - 9

12.5	Girl	5	5	2	V Ga	0.20	> 30

12.5	Boy	2	2	1	C	1.00	> 30

12.5	Girl	9	8	3	C V Ph	1.00	< 1

13	Boy	1	0	2	V L	0.22	< 1

14.5	Boy	2	2	1	O	1.00	< 1

14.5	Boy	4	3	1	V	0.50	1 - 9

15	Boy	5	4	2	C L	1.00	10 - 30

* V - Valproic acid; C - Carbamazepine; O - Oxcarbazepine; L - Lamotrigine; T - Topiramate; Cl - Clobazam; Et - Ethosuximide; Ga - Gabapentin; Ph - Phenobarbital

### Psychological measures

Parents of children completed the Health-Related Quality of Life in Childhood Epilepsy Questionnaire (QOLCE; [20]; Polish version [22]), a reliable epilepsy-specific parental questionnaire for children aged 4-18 years. The original scale was validated in Australia as well as in USA and its high reliability (Cronbach's alpha 0.93 and 0.92 accordingly) as well as good validity were confirmed [20,23]. The Polish version was adapted in a group of 87 children with epilepsy aged 4-18 years and it retain psychometric values of the original, with very high reliability (Cronbach's alpha 0.97) as well as good validity [22]. The scale contains 91 items, measuring an overall QOL, with two generic single-item subscales (general health item and QOL item) and 14 subscales, falling into five domains: physical function (physical restrictions, energy/fatigue), cognitive function (attention/concentration, memory, language, other cognitive), emotional well-being (depression, anxiety, control/helplessness, self-esteem), social function (social interactions, social activities, stigma item) and behavior. The parental judgment of their child's poor functioning is reflected by the lower score in overall scale as well as in any of 16 sub-domains. The Polish translation was approved by the authors of the original scale and its high reliability and validity were demonstrated.

### Procedure

During a visit in one of the specialist outpatient clinics, the child's parent/caregiver was asked to complete the QOLCE while the investigator was taking care of a child in a neighboring room. Prior to the interview, each child received an evaluation by a pediatric neurologist and neuropsychologist. As a part of the neurological evaluation each child underwent an EEG examination that was interpreted by a trained clinician. In addition, 23 out of 31 children underwent cranial computed tomography or magnetic resonance imaging.

### Statistical analysis

To test the main hypothesis, we conducted correlation analysis between lateralization and reduced QOL (overall QOL < 60; non-parametric Spearman's correlation). Two-sample t-tests for all sub-scales and domains were employed to describe the profile of impairments in the right- over the left-hemisphere group. Furthermore, left- and right-hemisphere groups were compared with regards to age, age of seizure onset, duration of disease, duration of medication, number and frequency of antiepileptic drugs (AED), number of hospitalizations per year, and average frequency of epileptiform discharges using two-sample t-tests. The level of significance was set according to p < 0.05 for all tests. All statistical analyses were performed with SPSS 14 software package (SPSS Inc., Chicago, IL).

## Results

Confirming the main hypothesis, lateralization and reduced overall QOL correlated significantly (Spearman's rho 0.361, p < 0.046). Children with right-hemispheric epileptiform discharges exhibited a lower overall QOL. Table 2 shows the comparisons for all domains, subscales, and items of the QOLCE. T-tests revealed that in particular the social functioning domain, the anxiety subscale as well as the quality-of-life, stigma, and general health items were rated as more compromised in right-hemispheric children. There was no statistically significant difference between the groups in terms of age or any of the clinical measures (all p > 0.13) and the gender was distributed similarly (6 girls and 9 boys in the left-hemispheric, 6 girls and 10 boys in the right-hemispheric group).

**Table 2:** Mean score and standard deviation in QOLCE subscales for left- and right-hemispheric groups.

	Left hemisphere	Right hemisphere	t (29)	Significance
Physical function	57.9 + 11.4	51.5 + 18.7	1.156	0.128

Physical restrictions	58.0 ± 15.0	47.2 ± 24.2	1.504	0.072

Energy/Fatigue	57.8 ± 12.0	55.8 ± 17.6	0.368	0.358

Cognitive function	58.6 + 19.8	55.8+26.4	0.337	0.369

Attention/Concentration	66.6 ± 23.7	53.3 ± 26.0	1.483	0.074

Memory	57.8 ± 20.4	58.1 ± 24.3	-0.030	0.488

Language	54.3 ± 23.7	59.0 ± 28.0	-0.501	0.310

Other cognitive	55.7 ± 20.1	52.8 ± 31.8	0.311	0.379

Emotional wellbeing	68.7 + 10.9	60.1 + 58.6	1.520	0.069

Depression	73.4 ± 13.6	65.0 ± 17.8	1.489	0.074

Anxiety	71.8 ± 19.9	58.6 ± 22.9	**1.726**	**0.048**

Control/Helplessness	61.7 ± 20.8	53.3 ± 22.1	1.088	0.143

Self-esteem	67.8 ± 13.2	63.3 ± 23.9	0.652	0.260

Social function	87.7 + 24.6	67.6 + 31.4	**1.987**	**0.028**

Social interactions	90.1 ± 33.9	71.7 ± 33.3	1.526	0.069

Social activities	79.2 ± 16.1	64.4 ± 29.6	**1.734**	**0.047**

Stigma item	93.7 ± 37.1	66.7 ± 34.9	**2.090**	**0.023**

Behavior	66.4 ± 15.1	58.9 ± 16.9	1.298	0.102

General-health item	59.4 ± 22.1	43.3 ± 27.5	**1.795**	**0.042**

QOL item	60.9 ± 18.2	38.3 ± 22.9	**3.055**	**0.002**

Overall QOL	67.2 ± 12.1	56.9 ± 19.7	**1.769**	**0.044**

Spearman's correlation analysis revealed lateralization effect. Significant differences for separate subscales between the groups, as revealed by two-sample t-test, are marked in bold.

## Discussion

We have demonstrated for the first time that in children lateralized epileptiform discharges have a differential impact on QOL. However, in contrast to adults [13,24], the right-hemispheric foci more often led to lower QOL. In particular, the lateralization affected emotional and social domains, including anxiety, social activities and stigma.

The studies concerning lateralization of emotions are not conclusive so far: the right hemisphere was suggested to dominate in the processing of emotional information. However the left hemisphere was considered to be more involved in the processing of positive emotions (for a review, see [25]). The association of negative emotional outcome with right-hemispheric seizures in adults is often explained by the "valence" hypothesis that associates negative emotions with the right hemisphere and positive with the left hemisphere [13]. The seizures or functional lesion in the left hemisphere could lead to deficits in processing of positive emotions, effecting the accentuation of dysphoric mood, whereas lesions of the left hemisphere would lead to deficits in processing of positive emotions. Kirsch [26] proposed an alternative view, linking psychosocial disturbances in epilepsy with subtle cognitive deficits. Those subclinical deficits, such as disturbed perception or interpretation of emotional prosody, influence social interactions and may lead to significant deficits in social functioning later in life.

Childhood is a critical period for developing cognitive and social skills. Therefore a disease that starts early in life may disrupt the acquisition of social cognition skills [27]. The earlier such deficits start, the more severe the consequences might be, leading to disturbed 'social cognition', that is the ability to interpret and understand oneself, others and the social world. Right temporal seizures often lead to subtle cognitive deficits described by Kirsch [26], such as disturbed recognition of fear [28] or problems with recognizing and identifying famous faces [29]. This model is in accordance with results of Cohen et al. [17], who demonstrated that children with right temporal focus had worse performance in recognizing emotional prosody than healthy controls. Such deficit in prosody recognition could be the cause of increased anxiety and stigma perception and disruption of social activities, leading in consequence to a lower general QOL. In all the children in our study, the disease occurred at least one year prior to the study, but usually much earlier, allowing the potential deficits to evoke the long-lasting effects on psychosocial functioning.

Ott et al. [30] demonstrated that even 66% of children with epilepsy can meet the criteria of a DSM-IV psychiatric diagnosis, although only about 30% of them receives any psychiatric treatment. These comorbidities have an impact on the life of child and the entire family [31]. Consequently, childhood-onset epilepsy has a long-term adverse impact on health-related QOL, even in adults who are seizure free, off medications for many years [32]. Camfield et al. [7] demonstrated in the study of 504 children with epilepsy, that most of the clinical parameters of treatment and seizure control, including remission, did not predict children's social functioning. It was observed that mood is the strongest predictor of QOL in epilepsy as compared to neuropsychological measures such as psychomotor speed, verbal memory, language, and cognitive inhibition [33].

Since patient's QOL may be improved by managing psychiatric and neuropsychological issues, even more than by reducing seizure frequency, Salpekar & Dunn [34] suggested, that the comprehensive treatment should take into account the etiology of psychiatric disturbances in epilepsy (e.g. pathophysiology of the brain, psychosocial stress of chronic epilepsy, medication side effects) and involve neurological, psychiatric as well as psychological modalities. Our results contribute to the understanding of developmental aspects of the link between brain pathophysiology and psychosocial functioning. Moreover, they create a basis for the development of more directed treatment strategies for young patients with unilateral seizures. Our study does not reveal the mechanism of the differences between left- and right-hemispheric groups. It points out, however, the importance of developmental aspects of the impact of seizures and allows for formulating directed hypotheses when planning future studies.

We found no statistically significant difference between the groups in their level of depression, which is often described in adults [11]. Since the questionnaires were filled by the parents of the children, not by the children themselves, one cannot exclude that parents did not notice the depressive features of their children. The possibility to miss signs of depression is the biggest disadvantage in the use of parental scales [35]. However, in a study by Mathiak et al. [18] there was also no higher depressiveness revealed in children with lateralized seizures, despite the use of self-report scales. Children in the left-hemispheric group attributed control over successful events in their lives to external sources, which is known to lead to depression later in life; the higher level of depressiveness found in adults with left-hemispheric focus may be a result of long-lasting, dysfunctional control attribution.

We decided not to assess the handedness of our subjects. While the relationship between handedness and hemispheric dominance appears to be strong in healthy right-handers (over 95%), it is much harder to draw any conclusions in left-handed or ambidextrous subjects, over 60% of them are reported to have left hemispheric dominance (for a review, see [36]). Hemispheric dominance correlates to a much lower extent with handedness in epilepsy, both in right- and left-handers [37]. Consequently, we decided not to exclude left-handed subjects from our study. To control reliably for hemispheric dominance we would need to apply invasive (e.g. Wada testing) or technically demanding and expensive (fMRI) techniques that were out of scope of our study. If some of our subjects had a reversed hemispheric dominance pattern, this would result in decrease of a statistical power of our results. It limits, therefore, conclusions concerning negative findings.

Although children with anatomical malformations were excluded from the study, 8 subjects did not undergo neuroimaging. However our group encompassed children with benign rolandic epilepsy. These patients may not be referred to brain imaging by their neurologist. Based on the clinical information and EEG alone we expected no anatomical malformations in those subjects [38]. If some of them had had bilateral or contralateral lesions that were not detected in the neuroimaging or not diagnosed due to a lack of clinical signs, it would have resulted in a reduced power of the study, just like undetected contralateral foci. As a consequence the effect sizes may be underestimated.

A limitation of the present study is the size of this strictly selected group of children with unilateral foci and no anatomical malformations. The statistical power does not allow for an assessment of lobar localization, which could provide a better insight into the neurobiological background of the observed disturbance. However, other studies involving child populations, fulfilling strict criteria of unilateral seizures and no structural lesions, encompass usually comparable or smaller groups only ([17]: 23 children; [39]: 12 children; [40]: 27 children; [41]: 12 children). Combining additional diagnostic methods, such as magnetoencephalography (MEG), functional magnetic resonance imaging (fMRI) and intensive video-EEG monitoring, in future studies on focus lateralization might help overcome the problem of a small group size and give a better insight into the understanding of the underling pathologies.

## Conclusions

Lateralization of epileptic focus can affect psychosocial functioning and lead to diminished QOL in children. Right-hemispheric foci may result in lower QOL, in particular in the domain of social functioning and emotions (anxiety). Focusing on the differences in QOL as a result of focus lateralization in development may have an important impact on treatment strategies and demands further studies. Taking into account developmental aspects in the course of epilepsy should facilitate understanding the complex influence of epileptic seizures on the brain structures. Childhood epilepsy can serve as a valuable neurobiological model to study the brain-behavior relationship.

## Competing interests

The authors declare that they have no competing interests.

## Authors' contributions

KAM conceived and designed the study, acquired the funding, participated in and supervised collection of data, performed statistical analysis, and drafted the manuscript. MŁ participated in collection of data and contributed to interpretation of results. KM participated in statistical analysis, contributed to interpretation of results and helped to draft the manuscript. KK participated in statistical analysis. TW participated in the design of the study. ES coordinated acquisition of clinical data and consulted clinical criteria of including patients. PO participated in the design and coordination of the study. All authors read and approved the final manuscript.

## Pre-publication history

The pre-publication history for this paper can be accessed here:

http://www.biomedcentral.com/1471-2377/10/69/prepub
